# Supplementary material for: Direct Photochemical Synthesis of Substituted Benzo[b]fluorenes
Source: Org Lett. 2024 Nov 25;26(48):10364–8. doi: 10.1021/acs.orglett.4c03978 (PMC11629382; doi:10.1021/acs.orglett.4c03978)
Supplement: Supplementary file 2 — ol4c03978_si_002.zip [file ol4c03978_si_002.zip › FID for Publication/20240711_RC-2-C-122A-D_01/plots/RC-2-C-122A-D_CARBON_20240711_01_plot01.pdf]

RC-2-C-122A-D

Location	**7**	Pulse sequence	**CARBON**	Temperature	**25**	Suboperator	**RuairiCrawford**
Date collected	**2024-07-11**	Solvent	**cdcl3**	Spectrometer	**Agilent-NMR-vnmrs500**	Operator	**MBaumann**

SAMPLE

date	Jul 11 2024
solvent	cdcl3
file	/home/data/MBaumann/newucd500/RuairiCrawford/2024/20240711_RC-2-C-122A-D_01/RC-2-C-122A-D_CARBON_20240711_01.fid

ACQUISITION

sw	31250.0
at	1.049
np	65536
fb	17000
bs	8
ss	40
d1	1.000
nt	1000
ct	1000

TRANSMITTER

tn	C13
sfrq	125.746
tof	1914.5
tpwr	58
pw	4.900

DECOUPLER

dn	H1
dof	0
dm	yyy
decwave	w
dpwr	41
dmf	12579

PRESATURATION

satmode	n
wet	n

SPECIAL

temp	25.0
gain	46
spin	0
hst	0.008
pw90	9.800
alfa	10.000

FLAGS

il	n
in	n
dp	y
hs	nn

PROCESSING

lb	0.50
fn	not used

DISPLAY

sp	-1794.5
wp	31250.0
rfl	1794.5
rfp	0
rp	23.2
lp	0

PLOT

wc	252
sc	8
vs	12483
th	5
ai cdc ph	

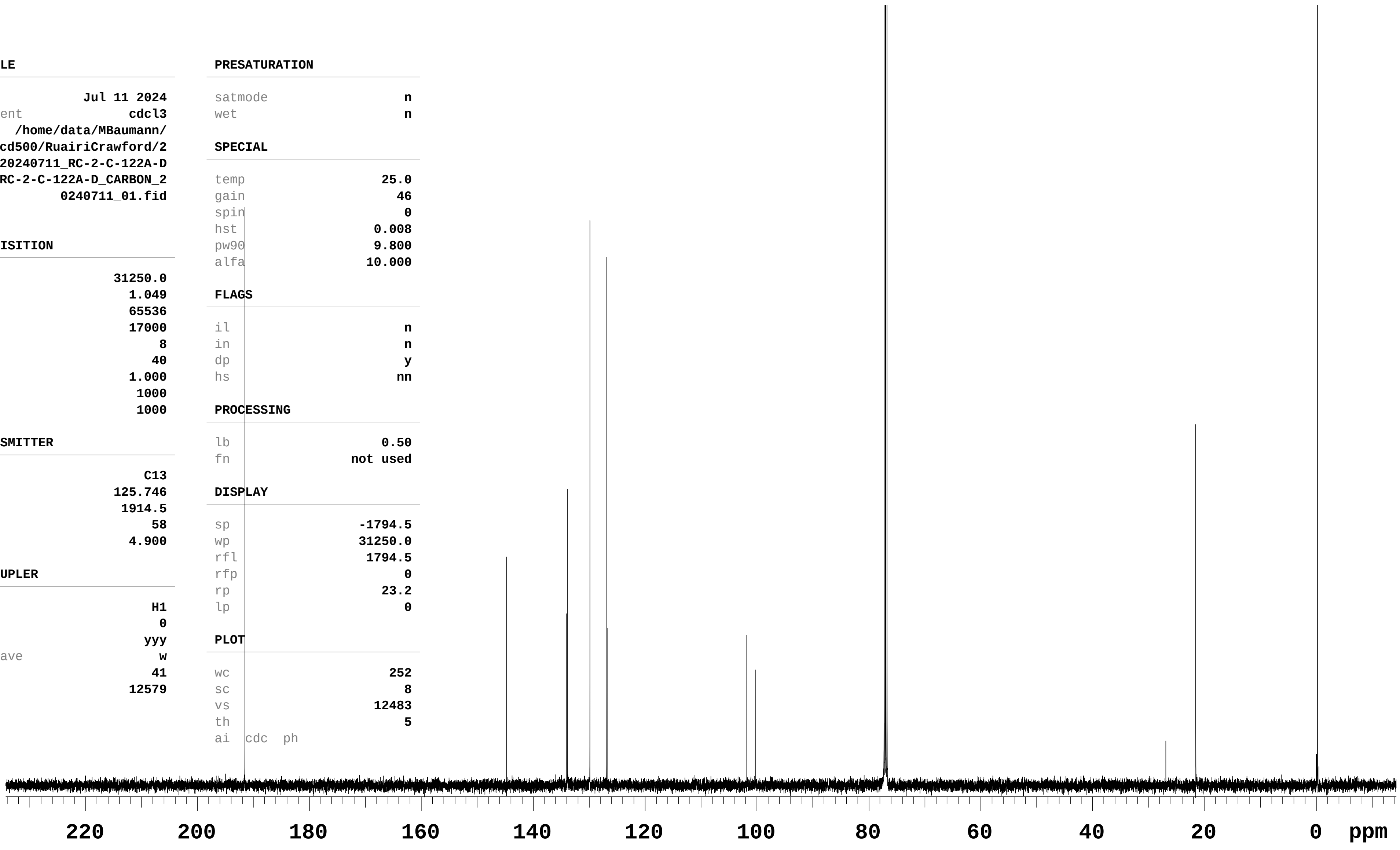

Plotname: RC-2-C-122A-D_CARBON_20240711_01_plot01

Data file /home/data/MBaumann/newucd500/RuairiCrawford/2024/20240711_RC-2-C-122A-D_01/RC-2-C-122A-D_CARBON_20240711_01.fid

Plot date 2024-07-11
